# Isoform-selective Hsp90 inhibition rescues model of hereditary open-angle glaucoma

**DOI:** 10.1038/s41598-017-18344-4

**Published:** 2017-12-20

**Authors:** Andrew R. Stothert, Amirthaa Suntharalingam, Xiaolan Tang, Vincent M. Crowley, Sanket J. Mishra, Jack M. Webster, Bryce A. Nordhues, Dustin J. E. Huard, Christopher L. Passaglia, Raquel L. Lieberman, Brian S. J. Blagg, Laura J. Blair, John Koren, Chad A. Dickey

**Affiliations:** 1 Department of Molecular Medicine and Byrd Alzheimer’s Research Institute, University of South Florida, Tampa, FL 33613 USA; 2 Department of Chemical & Biomedical Engineering, College of Engineering, University of South Florida, Tampa, FL 33613 USA; 3 Department of Medicinal Chemistry, The University of Kansas, Lawrence, KS 66045 USA; 4 School of Chemistry & Biochemistry, Georgia Institute of Technology, Atlanta, GA 30332 USA

## Abstract

The heat shock protein 90 (Hsp90) family of molecular chaperones regulates protein homeostasis, folding, and degradation. The ER-resident Hsp90 isoform, glucose-regulated protein 94 (Grp94), promotes the aggregation of mutant forms of myocilin, a protein associated with primary open-angle glaucoma. While inhibition of Grp94 promotes the degradation of mutant myocilin *in vitro*, to date no Grp94-selective inhibitors have been investigated *in vivo*. Here, a Grp94-selective inhibitor facilitated mutant myocilin degradation and rescued phenotypes in a transgenic mouse model of hereditary primary open-angle glaucoma. Ocular toxicities previously associated with pan-Hsp90 inhibitors were not evident with our Grp94-selective inhibitor, 4-Br-BnIm. Our study suggests that selective inhibition of a distinct Hsp90 family member holds translational promise for ocular and other diseases associated with cell stress and protein misfolding.

## Introduction

Primary open-angle glaucoma (POAG), a degenerative optic neuropathy characterized by elevated intraocular pressure (IOP), is a leading cause of irreversible vision loss and blindness^1–3^. Mutations in the myocilin gene (*MYOC*) are the most common genetic causes of glaucoma, accounting for 3–5% of POAG and up to 35% of Juvenile open-angle glaucoma (JOAG)^4–7^. More than 70 mutations in *MYOC*, predominantly within its five-bladed β-propeller olfactomedin (OLF) domain, have been identified^8,9^. These mutations prompt a toxic gain-of-function, resulting in myocilin aggregation within the endoplasmic reticulum (ER) of trabecular meshwork (TM) cells^10–13^. Myocilin misfolding triggers a cascade of events leading to TM cell degeneration, increased IOP, progressive retinal ganglion cell (RGC) death, and axonal degeneration of the optic nerve^3,6,9^. Mice over-expressing the Y437H human *MYOC* mutant (Tg-MYOC^Y437H^) recapitulate the pathophysiology of hereditary POAG, including reduced aqueous humor outflow, elevated IOP and RGC death and dysfunction culminating in vision loss^14^.

Our previous studies demonstrated that the ER-resident heat shock protein 90 (Hsp90) isoform, glucose regulated protein 94 (Grp94), recognizes mutant *MYOC* in an aberrant interaction that subverts proper degradation of the mutant protein^15,16^. Inhibition of Grp94, with either a pan-Hsp90 inhibitor, a Grp94-selective inhibitor, or Grp94 knockdown cleared mutant *MYOC* in cell culture models^16^. Pan-Hsp90 inhibitors, while capable of inhibiting Grp94, are poor therapeutic options for POAG due to ocular toxicities observed in clinical trials and animal studies^17,18^. Unlike Grp94, cytosolic Hsp90 isoforms regulate and maintain a large number of proteins essential for cellular survival; allowing pan-Hsp90 inhibition to exhibit a wide range of undesired effects^19–23^. Our findings suggest a selective inhibition of Grp94 would be a unique strategy to treat mutant *MYOC*-induced POAG. Directly inhibiting Grp94 and clearing a molecular driver of POAG pathology represents a therapeutic strategy distinct from many current glaucoma therapeutics, which attempt to alter either the production or the outflow of aqueous humor^1,3^.

Here, we demonstrate the *in vivo* efficacy of a Grp94-selective inhibitor in a well-characterized transgenic mouse model of familial POAG^14^. Selective inhibition of Grp94 reduced intracellular levels of mutant myocilin. Concomitantly, myocilin-associated glaucomatous phenotypes, including elevated IOP and RGC function, were rescued. This is the first demonstration of *in vivo* efficacy for a Grp94-selective inhibitor. Additionally, this is the first potential therapeutic agent for the treatment of POAG and JOAG which acts by clearing mutant myocilin.

## Results

### 4-Br-BnIm binds within the ATP-binding pocket of Grp94

**Figure 1:**
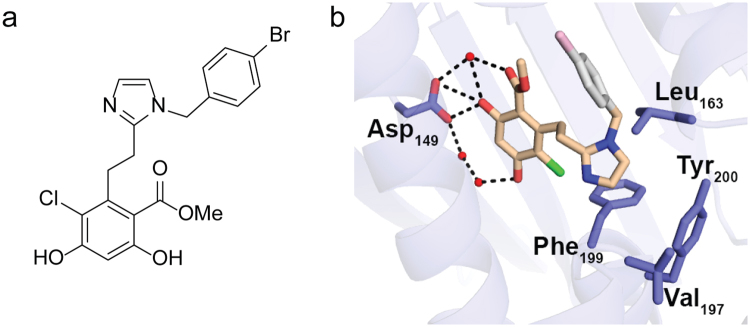
4-Br-BnIm interacts with the ATP-binding pocket of Grp94. (**a**) Chemical structure of Grp94-selective inhibitor, 4-Br-BnIm. (**b**) Crystal structure of the N-terminal domain of Grp94 in complex with 4-Br-BnIm. 4-Br-BnIm bound in the ATP binding pocket of the Grp94 NΔ41 construct, based on a 2.7 Å resolution crystal structure (see Supplementary Table 1). Grey: not observed in electron density. Black dash: H-bonding interactions. Red ball: modeled water molecules. Green: chloride substituent.

X-ray crystallography was used to determine interactions between 4-Br-BnIm (Fig. 1a) and the Grp94 N-terminal ATP-binding site. The crystal structure of the N-terminal domain of Grp94 in complex with 4-Br-BnIm (Extended Data Table 1), reveals a binding pose in which the resorcinol ring is anchored into the ATP binding pocket via direct and water-mediated hydrogen bonding interactions with Asp149 (Fig. 1b). Additional interactions included an apparent electrostatic pairing between Asn107 and the chloride-substituent of the resorcinol ring. Electron density is not readily visible for the brominated benzene substituent of 4-Br-BnIm and the adjacent Grp94 loop (residues 165–170) that caps the ATP binding pocket, suggesting that several conformations of 4-Br-BnIm may be present.

### Distribution of 4-Br-BnIm in mouse eye

We assessed the retention of 4-Br-BnIm in the eye to generate an *in vivo* treatment strategy. Following a single application of 100 µM 4-Br-BnIm (10 µL eye drop), treated mice were sacrificed, and whole eyes were collected for high-performance liquid chromatography (HPLC) analysis. Approximately 13% of the single administration (61.3 ng of 466ng delivered) was retained (Table 1 and Extended Data Fig. 1). Next, 100 µM 4-Br-BnIm eye drops were applied once daily for seven days. Treated mice were sacrificed 24 hours after the final administration. Treated eyes were enucleated and dissected into anterior and posterior segments for HPLC analysis. Calculated concentration of 4-Br-BnIm in the whole eye was 4.3 µM, which was evenly distributed between the anterior and posterior segments (Table 1). Retention of 4.3%, down from 13% of the single administration, suggested that 4-Br-BnIm was not accumulating in the eye. We selected a regimen of a once daily dose of 300 µM 4-Br-BnIm for our *in vivo* studies, which we estimate will maintain an eye concentration of ~12 µM.

**Table 1:** 4-Br-BnIm topical delivery to the eye.

Treatment	Tissue	4-Br-BnIm retained (ng)	Concentration in eye^††^ (µM)
Single 100 µM dose^†^	Whole eye	61.3 ± 7.9	6.59 ± 0.84
7 daily 100 µM doses	Whole eye	40.0 ± 1.3	4.29 ± 0.14
	Anterior eye	19.8 ± 1.5	4.25 ± 0.33
	Posterior eye	20.2 ± 1.5	4.33 ± 0.33

^†^One dose = 466 ng of 4-Br-BnIm in a 10 µl drop.

^††^Concentration in eye based on a 20 mm^3^ eye volume.

All values expressed ± SD (n = 7).

### Grp94-selective inhibition alleviates a glaucoma phenotype *in vivo*

**Figure 2:**
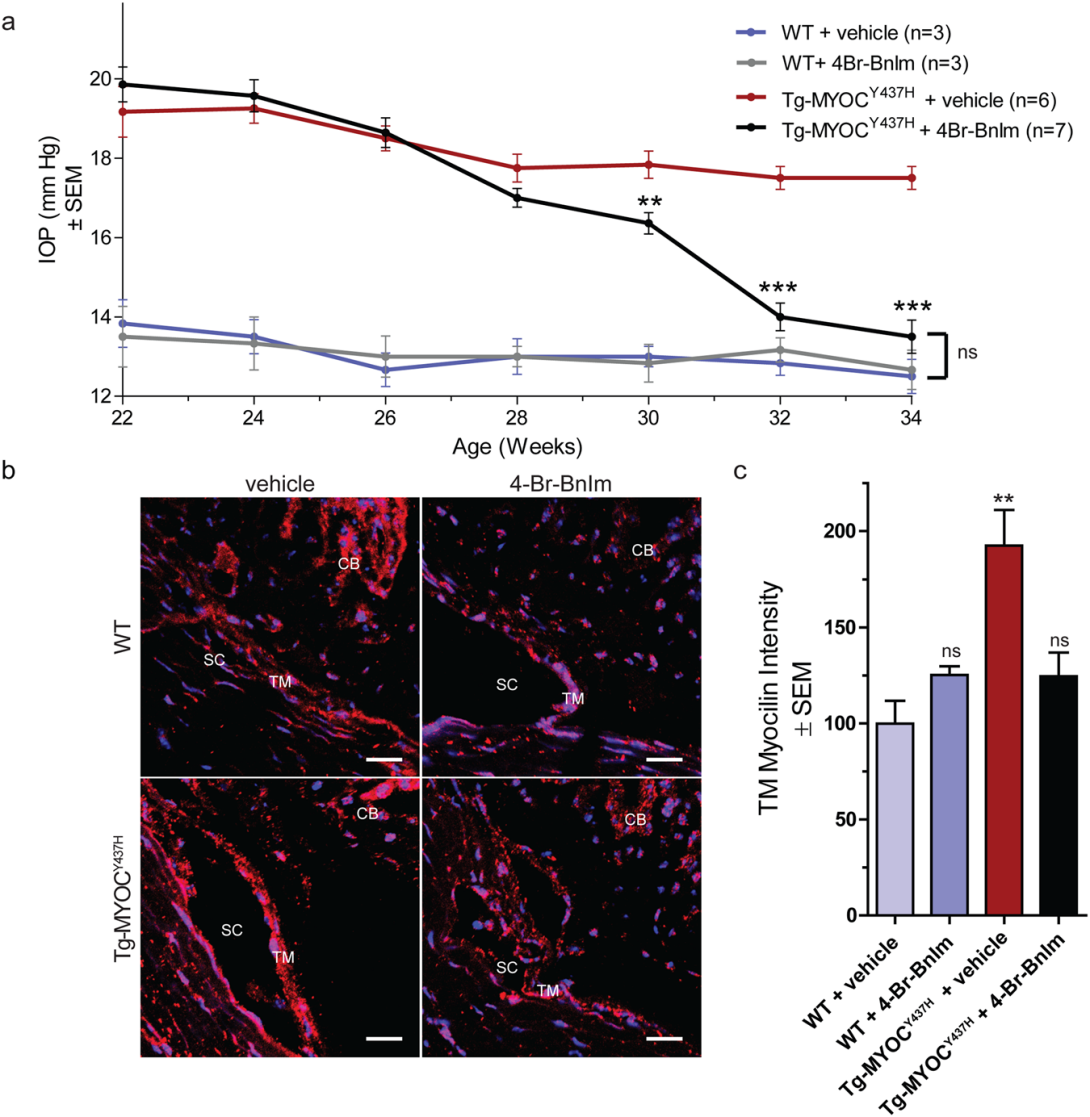
4-Br-BnIm rescued IOP and reduced myocilin accumulation in Tg-MYOC^Y437H^ mice. (**a**) IOP was measured biweekly over 12 weeks of treatment with vehicle or 4-Br-BnIm. Statistical analysis was carried out using one-way ANOVA with Bonferroni post-hoc test. *P < 0.05, ***P < 0.001. WT + vehicle (n = 3) WT + 4-Br-BnIm (n = 3), Tg-MYOC^Y437H^ + vehicle (n = 6), Tg-MYOC^Y437H^ + 4-Br-BnIm (n = 7). (**b**) Representative images depicting myocilin accumulation (red) in the trabecular meshwork (TM). TM, Schlemm’s canal (SC) and ciliary body (CB) are labeled. DAPI is used as a nuclear counterstain (blue). Scale Bar = 20 µm. (**c**) Quantification of myocilin levels. Statistical analysis was carried out using one-way ANOVA with Bonferroni post-hoc test. **P < 0.01, F = 5.77, df = 14. WT + vehicle (n = 2) WT + 4-Br-BnIm (n = 3), Tg-MYOC^Y437H^ + vehicle (n = 5), Tg-MYOC^Y437H^ + 4-Br-BnIm (n = 5).

Based on previous characterization of the Tg-MYOC^Y437H^ mouse model, POAG phenotype in the Tg-MYOC^Y437H^ appear at three months of age^14^. Hence, four-month old Tg-MYOC^Y437H^ and WT littermates were treated with 300 µM 4-Br-BnIm or 0.9% saline (vehicle) eye drops once daily for 12 weeks and diurnal IOP measurements were recorded biweekly on each eye. Grp94 inhibition reduced the IOP of Tg-MYOC^Y437H^ mice to levels significantly lower than vehicle treated Tg-MYOC^Y437H^ mice after 8 weeks of treatment, and to levels indistinguishable from WT after 10 weeks of treatment (Fig. 2a). Importantly, no changes in IOP were observed in WT mice following Grp94 inhibition, indicating that Grp94 is not an IOP reducing agent. Reduced IOP in Grp94 inhibitor-treated Tg-MYOC^Y437H^ mice was accompanied by clearance of mutant myocilin within TM cells (Fig. 2b,c). Levels of myocilin in WT mice were not affected This result is consistent with our previous cell culture studies demonstrating Grp94 selectivity for mutant myocilin but not wild-type myocilin^16^.

### Topical ocular Grp94 inhibition stops progressive RGC deficits in Tg-MYOC^Y437H^ mice

**Figure 3:**
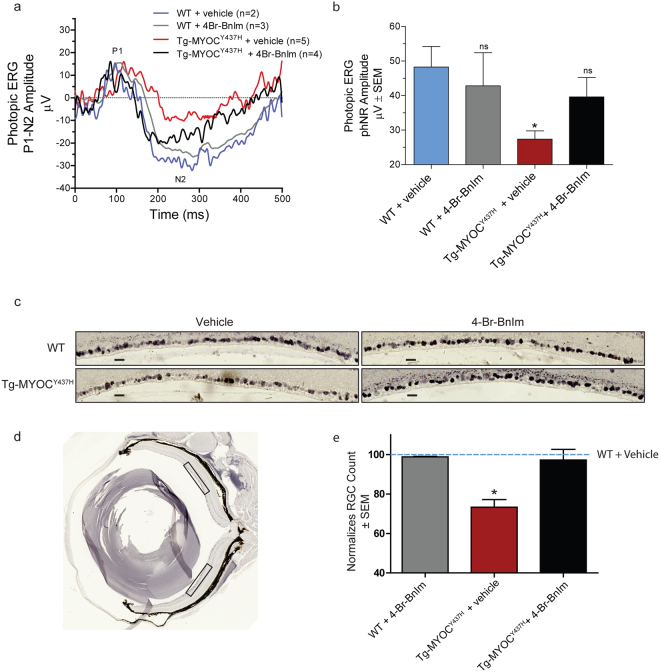
4-Br-BnIm reversed the light-adapted response phenotype in transgenic Tg-MYOC^Y437H^ mice and protects RGCs. (**a**) Light-adapted electroretinography (LA-ERG) tracings of WT and Tg-MYOCY437H mice without and with 4-Br-BnIm treatment. (**b**) Quantification of the PhNR from LA-ERG tracings. Statistical analysis was carried out using one-way ANOVA with Bonferroni post-hoc test. *P < 0.05, F = 2.48, df = 27. n values represent individual mice. WT + vehicle (n = 2), WT + 4-Br-BnIm (n = 3), Tg-MYOC^Y437H^ + vehicle (n = 5), Tg-MYOC^Y437H^ + 4-Br-BnIm (n = 4). (**c**) Representative images showing DAB stained NeuN positive cells in a 350 µm length of RGC layer from the midperipheral region of the retina from each treatment group. Scale bars = 20 µm. (**d**) A representative whole eye section from a WT vehicle mouse, boxes identify the region of retina used in the quantitation of RGC density. (**e**) Quantification of RGC density normalized to a WT vehicle treated mouse. Statistical analysis was carried out using two-tailed unpaired t-test. *P < 0.05, t = 3.63, df = 4. WT + 4-Br-BnIm (n = 3), Tg-MYOC^Y437H^ + vehicle (n = 3), Tg-MYOC^Y437H^ + 4-Br-BnIm (n = 3).

Chronic elevated IOP leads to a progressive decline in RGC function and RGC loss^1,9,14^. Light-adapted electroretinography (LA-ERG) was used to measure the photopic negative response (PhNR), a sensitive measure for ganglion cell function. Tg-MYOC^Y437H^ demonstrated reduced PhNR amplitude compared to WT mice (Fig. 3a,b). Selective inhibition of Grp94 in Tg-MYOC^Y437H^ mice restored the PhNR deficits (Fig. 3b). Additionally, Tg-MYOC^Y437H^ mice have reduced NeuN positive RGC counts in the ganglion cell layer compared to WT mice. This deficit was ameliorated in Tg-MYOC^Y437H^ mice following reduction of mutant myocilin by Grp94-selective inhibition (Fig. 3c–e). No significant differences were observed in both measures of retinal health, ERG and NeuN-positive RGC count in WT mice treated with and without 4-Br-BnIm. Furthermore, these results show that selective inhibition of Grp94 did not elicit ocular toxicities previously observed with pan-Hsp90 inhibition.

### 4-Br-BnIm does not induce a heat shock response

**Figure 4:**
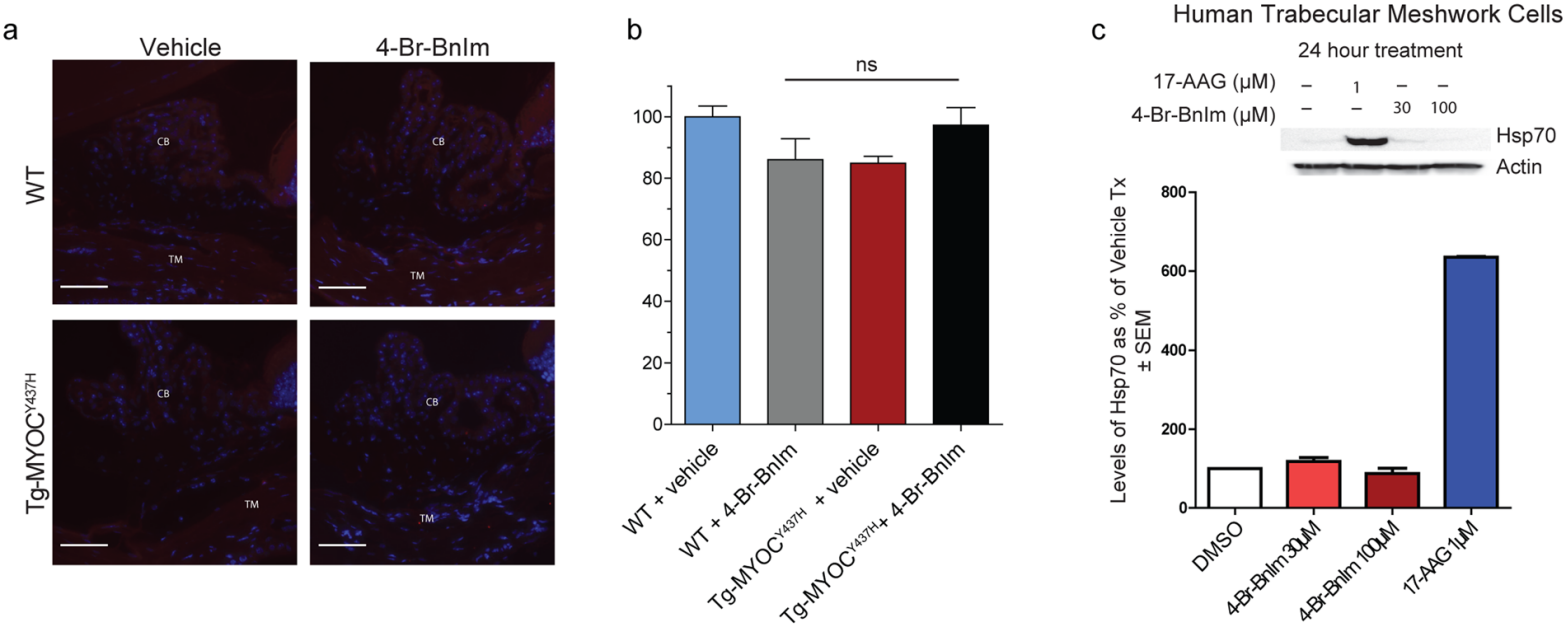
4-Br-BnIm does not induce Hsp70 in Tg-MYOC^Y437H^ mice. (**a**) Representative images depicting Hsp70 levels (red fluorescence), as observed by fluorescent immunostaining and multiphoton microscopy, in the trabecular meshwork (TM) of mouse tissue. TM and ciliary body (CB) are labeled. DAPI is used as a nuclear counterstain (blue). Scale Bar = 50 µm. (**b**) Quantification of Hsp70 levels normalized to WT vehicle-treated controls. Error bars represent mean ± SEM. Eyes assessed: WT + vehicle (n = 2), WT + 4-Br-BnIm (n = 3), Tg-MYOC^Y437H^ + vehicle (n = 7), Tg-MYOC^Y437H^ + 4-Br-BnIm (n = 4). No significant difference was observed between groups as determined by one-way ANOVA analysis, F = 2.8, df = 15. (**c**) Western Blot analysis and quantitation of Hsp70 levels following vehicle, 17-AAG, and 4-Br-BnIm treatment to HTM cells.

Induction of the heat shock response resulting in an increased expression of the 70 kDa heat shock protein (Hsp70) is a hallmark of pan-Hsp90 inhibition^24,25^. Hsp70 levels were assessed via immunofluorescence imaging of TM cells from both WT and Tg-MYOC^Y437H^ mice treated with and without 4-Br-BnIm. Consistent with our previous *in vitro* results^16^, no significant differences were observed in Hsp70 levels following treatment with 4-Br-BnIm in either WT or transgenic groups (Fig. 4a,b). As a comparison, human trabecular meshwork (HTM) cells were treated with either 1 µM of the pan-Hsp90 inhibitor 17-AAG, or one of two concentrations (30 and 100 µM) of the Grp94-selective inhibitor 4-Br-BnIm for twenty-four hours. Lysis and Western Blot analysis of the treated HTM cells revealed a 600% increase in Hsp70 levels following treatment with the pan-Hsp90 inhibitor, 17-AAG. Minimal changes to Hsp70 levels were observed at either concentration of 4-Br-BnIm (Fig. 4c).

## Discussion

This work extends our previous studies of Grp94 and mutant myocilin to a well-characterized transgenic mouse model of hereditary open-angle glaucoma^14^. Compared to WT myocilin, which is secreted from TM cells, mutant myocilin variants are sequestered within the ER, where it forms a non-productive interaction with Grp94 that precludes clearance and leads to toxic accumulation^13^. Previous efforts in targeting mutant myocilin have focused on increasing mutant myocilin secretion, thus restoring function^11,26–28^ or enabling degradation via phagocytosis activities of TM cells^29^.

Since myocilin knockout mice^30^ and individuals harboring distinct truncated forms of myocilin protein^31^ do not develop glaucoma, the accumulation mutant myocilin represents a toxic gain of function. Grp94, the ER resident member of the Hsp90 family of molecular chaperones, stabilizes mutant myocilin. Here, we demonstrate, for the first time *in vivo*, that a Grp94-selective small molecule, 4-Br-BnIm, cleared mutant myocilin, leading to a reduction in glaucomatous phenotypes of IOP and rescued RGC dysfunction. The detrimental effects commonly associated with pan-Hsp90 inhibition, including retinal toxicities^17,18,32^ were not observed following treatment with 4-Br-BnIm. However, due to the low number of animals examined in these studies, additional experiments will be necessary on animal cohorts with more robust numbers to thoroughly assess the translatability of this therapeutic strategy. Despite this, these data suggest that reducing mutant myocilin levels by inhibiting Grp94 is a viable therapeutic strategy for hereditary POAG and should be well tolerated.

4-Br-BnIm represents one of several small molecule Grp94-selective inhibitors under development to overcome the liabilities associated with pan- Hsp90 inhibition^19–22^. Specifically, the nucleotide binding pocket within the Grp94 N-terminal domain differs from other Hsp90 isoforms by an insertion of five amino acids. Thus, Grp94 harbors a unique, largely hydrophobic, pocket that can be exploited in inhibitor design. Indeed, selectivity has been improved with iterative structure activity relationship studies^33–35^. The structure of the N-terminal domain of Grp94 in complex with 4-Br-BnIm revealed a pose in which the bromo-benzyl moiety was not locked in a single conformation. Thus, we infer that this region of the inhibitor is sampling multiple binding modes, including the novel hydrophobic pocket targeted in the structure-guided approach. Future work will further refine structure-activity relationships for Grp94 selectivity across chaperone and non-chaperone off-targets. Mechanistically, inhibition of nucleotide binding domain of Grp94 inhibits the apparently irreversible binding of mutant myocilin, enabling degradation via autophagy instead of continually failing at ERAD^15^. This process spares TM cells from apoptosis, thus TM tissue integrity and IOP should be unaffected even if mutant myocilin is being translated.

Selective inhibition of Grp94 may also be translatable into a clinical treatment option for POAG more generally. Few specific molecular underpinnings of glaucoma are known^1–3^ but ER stress may be globally relevant^14,36^. Besides the fact that ER stress is an outcome of mutant myocilin accumulation^14,15,37,38^, ER stress was found to be the causative factor for RGC death following exposure to elevated IOP^39^. ER stress was also elevated in a mouse model of steroid-induced glaucoma following topical application of dexamethasone^40^, which is known to induce expression of wild-type myocilin^41,42^ but not directly implicated in this secondary glaucoma subtype^43^. In summary, reduction of ER stress, regardless of its origin, may be a promising interventional strategy to treat both the cause and effect of glaucoma pathology.

## Methods

### 4-Br-BnIm synthesis and crystal structure determination with the N-terminal domain of Grp94

4-Br-BnIm was synthesized as previously described^34^. A truncated N-terminal domain of Grp94 (NΔ41, residues 69–337; residues 287–327 replaced by GGGG) was expressed and purified as previously described^34^. Crystals of apo-NΔ41 were grown by the hanging drop method with equilibration of the protein (at 15 mg/mL) against a reservoir solution comprised of 35% PEG400, 100 mM Tris at pH 7.5, and 80 mM MgCl_2_. Crystals were harvested and subsequently soaked for 30 minutes in a mother liquor solution containing 20 mM 4-Br-BnIm, which was diluted from a 100 mM stock solution prepared in DMSO. Then crystals were treated with glycerol (20% v/v) and cryo-cooled in liquid nitrogen. X-ray diffraction data were collected at the Advanced Photon Source, Argonne National Labs Southeast Region Collaborative Access Team (SER-CAT) beamline 22-BM. XDS/XSCALE were used to process the data^44^. The structure was solved by molecular replacement in Phaser^45^ using the polypeptide chain PDB ID 2FGD as the search model. Iterative model building and refinement were performed using Coot^46^ and Phenix.refine^47^. Models of PEG400 and glycerol ligands were generated with corresponding SMILES strings, and additional restraints for these ligands were prepared with eLBOW in Phenix^47^. A model for 4-Br-BnIm was prepared via the PRODRG2 server^48^. The structure was deposited to the PDB with accession code 5TTZ.

### Animal Husbandry

Mice were housed and bred at the University of South Florida Byrd Alzheimer’s Institute. The transgenic-Y437H mouse model (Tg-MYOCY437H) was a generous gift from Val Sheffield at the University of Iowa^14,49^. Heterozygous Tg mice were bred on the C57BL/6 background to produce homozygous and WT littermates for studies, as well as heterozygous mice for breeding. Genotyping for the transgene was performed as previously described^14,49^. All animal procedures performed in this study were in compliance with the ARVO Statement for the Use of Animals in Ophthalmic and Vision Research and were approved by the University of South Florida Institutional Animal Care and Use Committee. A cohort of 19 animals from two separate litters were used for this study. Total number of mice at the start of the experiment WT + vehicle (n = 3) WT + 4-Br-BnIm (n = 3), Tg + vehicle (n = 6), Tg + 4-Br-BnIm (n = 7).

### Topical Ocular Delivery of 4-Br-BnIm

4-Br-BnIm treatment dose was decided based on HPLC analysis. For the single dose analysis of eye permeability, WT C57BL/6 mice were treated with a topically administered drop of 4-Br-BnIm (~10 µL) at 100 µM concentration for 1 minute before it was irrigated off with sterile PBS and mice were then immediately euthanized. To evaluate delivery over time, 4-Br-BnIm was delivered once daily for 7 days and mice were euthanized 24 hours after the last dose. Isolated flash frozen eye samples were homogenized by vortexing with glass beads. Organics were extracted using EtOAc, which was then removed by evaporation and samples were diluted in MeCN/H2O (1:1) with 1% MeOH for HPLC analysis. HPLC Conditions: Agilent 1100 series quaternary pump, Agilent C-18 column (4.6 × 150 mm, 5 µm), 1.0 mL/min, 50% MeCN/50% H_2_O, detection at 209 nm, R_t_ = 5.3 min. For treatment studies, 4-Br-BnIm was solubilized in DMSO and diluted in sterile saline. The final concentration of DMSO was 1%. Once a day, mice were restrained and 1 drop (~10 µL) of drug at 300 µM was applied topically. The drop was allowed to sit on the eye for 1 minute before the mouse was returned to its cage. Mice were treated 1x/day for 12 weeks.

### IOP Measurements

IOP levels in the mouse eye were obtained using the Icare TonoLab rebound tonometer and their guidelines were followed (Icare, Finland). Biweekly, mice were anesthetized with 3–4% isoflurane in oxygen. Once induced, mice were placed in a tube restraint, and IOP measurements were taken for each eye. Animals were anesthetized for no more than 2 minutes during the IOP measurement process. Measurements were taken at same time of day to control for fluctuations in IOP levels which occur throughout the day. All mice were housed in the same housing room in the University of South Florida Byrd Alzheimer’s Institute. IOP measurements were taken in the same procedure room in the University of South Florida Byrd Alzheimer’s Institute.

### Electroretinograms

Electroretinograms (ERGs) were recorded from both eyes of each mouse post-treatment in a soundproof booth. Prior to ERG recordings, mice were anesthetized using a Ketamine (100 mg/kg) and Acepromazine (1 mg/kg) mixture delivered via intraperitoneal (IP) injection. Mouse depth of anesthesia was monitored throughout the experiment via paw pinches and heart rate recordings with subdermal ECG electrodes, and additional anesthesia was given as needed. A custom ring-shaped gold electrode was placed on the corneal limbus of each eye. Platinum needles (Natus Neurology Inc., Warwick, RI) inserted in the temples and tail served as references and ground electrodes, respectively. Recorded ERG signals were differentially amplified (2000x) and filtered (0.1–1000 Hz) by a multi-channel bio-amplifier (Xcell-3 × 4, FHC Inc., Bowdoin, ME) and digitized at 1000 Hz. Light stimuli were produced by a green LED (peak wavelength: 520 nm, Vishay TLCTG5800, Newark Electronics, Palatine, IL) with an 8° emittance angle positioned 1 cm from the cornea and aligned to the optic axis of each eye. ERGs were recorded for a series of 15–50 brief (10ms) full-field flashes (1.32–0.132 log cd·s/m^2^) delivered to both eyes simultaneously. Full-field ERGs were recorded from the light-adapted (photopic) eyes. Flashes were separated by a 3 second interval to allow for recovery from prior flashes in the sequence. ERG signals were quantified in terms of the photopic negative response, measured as the difference in amplitude from the maximum peak of the b-wave (between 50–150 ms) to the minimum value of the PhNR (200–300 ms). Statistical analysis and graphs were generated with GraphPad Prism 5.0 software.

### Eye enucleation

Mice were euthanized with a 0.2% Somnasol (50 mg/kg) in saline solution. Eyes were gently removed from the skull preserving the morphology of the eye globe. Once removed from the skull, eyes were either flash frozen for PK studies or fixed in Davidson’s fixative (1:3:2:3, glacial acetic acid: 95% ethanol, 10% neutral buffered formalin, distilled water) solution for histology.

### Histological processing of mouse eye tissue

Enucleated mouse eyes were immediately fixed in Davidson’s Solution for 48 hours. Then eyes were placed in 10% neutral buffered formalin for shipping to HistoWiz Inc. (Brooklyn, NY, USA) where tissue was processed, paraffin embedded, sectioned (10 µm thick), and mounted on slides. Slides were placed in a glass slide rack, and deparaffinized/rehydrated using the following steps: 2 × 3 min wash in xylenes (Millipore, #XX0060-4), 1 × 3 min wash in 1:1 Xylenes:100% ethanol, 2 × 3 min wash in 100% ethanol, 1 × 3 min wash in 95% ethanol, 1 × 3 min wash in 75% ethanol, 1 × 3 min wash in 50% ethanol, keep in ddH_2_O until antigen retrieval. After rehydration, at no point were the slides/tissue allowed to dry out. Slides for myocilin and Hsp70 staining were processed for heat-induced antigen retrieval as follows repeated three times. Slides were submersed in a Sodium Citrate Buffer (10 mM Sodium Citrate, 0.05% Tween 20, ph 6.0) in a plastic coplin jar placed in a water bath and, microwaved in 2 minute increments until boiling, replacing water and sodium citrate butter after each heating cycle. Endogenous peroxidase activity was quenched with 3% H_2_O_2_ and tissue was blocked and permeabilized with 1% Triton X-100 in PBS containing 4% goat serum.

### Immunofluorescent tissue staining

Eye tissue sections was incubated in 4% goat serum containing anti-myocilin (Santa Cruz, Dallas, TX, 1:50, # sc-21243) or anti-Hsp70 (Enzo Life Sciences, Farmingdale, NY, 1:100,# ADI-SPA-810-D) primary antibodies overnight. The following day, sections underwent PBS washing, and tissue was incubated with Alexa Fluor 594 (Invitrogen, Grand Island, NY; 1:500) secondary antibody for 2 hours and washed in PBS. Sections were then stained with DAPI for 20 minutes, washed and cover-slipped using ProLong Gold Antifade Reagent (Invitrogen).

### Chromogenic tissue staining

NeuN staining of tissue was done as previously described^50^. Briefly, Slides were incubated overnight with biotin conjugated anti-NeuN (Millipore, Billerica, MA, 1:250,# MAB377B) in 4% (v/v) goat serum. The next day, slides were washed three times with PBS and proceeded with DAB (3,3-diaminobenzidine)/Nickel stain. Slides were dehydrated and eye sections were cover-slipped using DPX mounting medium (Sigma, St. Louis, MO).

### Tissue imaging, quantification, and analysis

Hsp70 stained eye sections were imaged using the Olympus FV1000 MPE Multiphoton Laser Scanning Microscope at 40x objective (scale bar = 50 µm). Myocilin stained eye sections were imaged using the Zeiss LSM 880 (Oberkochen, Germany) at 20x objective (scale bar = 20 µm). NeuN stained eye sections were imaged using the Carl Zeiss AxioImager.Z1 microscope (Oberkochen, Germany) using a 20x dry Zeiss EC Plan-Neofluar objective (scale bar = 20 µM). Hsp70 and myocilin fluorescent intensity was quantified using ImageJ analysis software (National Institutes of Health). For whole eye sections, each mouse had two TM regions analyzed using five representative regions of interest. These values were then averaged to determine average myocilin or HSP70 level in each eye. RGC density was measured by number of cells unambiguously positive for NeuN within the RGC layer in the mid-peripheral retina which was determined via a certain distance from the optic disc^51^. For each eye section two ROIs were counted and the mean was used as an RGC density for each eye.

### Western Blotting

Human trabecular meshwork cells were treated for 24 hours with a vehicle (DMSO), 1 µM 17-AAG, or 4-Br-BnIm at 30 or 100 µM. Cells were lysed in RIPA buffer. Lysates were separated by SDS-PAGE and transferred to a PVDF membrane. Hsp70 (Enzo, 1:1000) and Actin (Sigma Aldrich, 1:3000) levels were assessed by addition of primary antibodies, species appropriate secondary antibodies (Southern Biotech, 1:1000), and exposed to ECL for chemiluminescent detection.

### Quantification and Statistical Analysis

Two-tailed student’s t-test, one-way ANOVA, and two-way ANOVA with Bonferroni’s post-hoc tests were used as detailed in the figure legends. Values were considered significant if p < 0.05. Graphs were generated using GraphPad Prism 5.0 analysis software. N-value in this study is depicted as number of animals or number of eyes where indicated.

### Data availability

The structure has been deposited in the Research Collaboratory for Structural Bioinformatics protein databank under PDB-ID: 5TTZ. All relevant data are available from the authors.

## Electronic supplementary material

Extended Data
