# Supplementary material for: Isoform-selective Hsp90 inhibition rescues model of hereditary open-angle glaucoma
Source: Sci Rep. 2017 Dec 20;7:17951. doi: 10.1038/s41598-017-18344-4 (PMC5738387; doi:10.1038/s41598-017-18344-4)
Supplement: Supplementary file 1 — Extended Data [file 41598_2017_18344_MOESM1_ESM.pdf]

Isoform-selective Hsp90 inhibition rescues model of hereditary open-angle glaucoma

Andrew R. Stothert[1†], Amirthaa Suntharalingam[1†], Xiaolan Tang[1,2], Vincent M. Crowley[3], Sanket J. Mishra[3], Jack M. Webster[1], Bryce A. Nordhues[1], Dustin J. E. Huard[4], Christopher Passaglia[2], Raquel L. Lieberman[4], Brian S. J. Blagg[3], Laura J. Blair[1*], John Koren III[1*], Chad A. Dickey[1‡]

Affiliations:

[1]Department of Molecular Medicine and Byrd Alzheimer's Research Institute, University of South Florida, Tampa, FL, 33613, USA,

[2]Department of Chemical & Biomedical Engineering, College of Engineering, University of South Florida, Tampa, FL, 33613, USA,

[3]Department of Medicinal Chemistry, The University of Kansas, Lawrence, KS, 66045, USA and

[4]School of Chemistry & Biochemistry, Georgia Institute of Technology, Atlanta, GA, 30332, USA

[†]These authors contributed equally to the development of the manuscript

[‡]Deceased November, 2016.

[*]Corresponding Authors:

Laura J. Blair

Assistant Professor

USF Health Byrd Institute

4001 E. Fletcher Ave., MDC 36

Tampa, FL 33613

(813)396-0639

lblair@health.usf.edu

John Koren III

Research Associate

USF Health Byrd Institute

4001 E. Fletcher Ave., MDC 36

Tampa, FL 33613

(813)396-0639

jkoren@health.usf.edu

Keywords: GRP94, glaucoma, myocilin, chaperones, Hsp90

Extended Data

Extended Data Figure 1: Representative HPLC traces used for quantification of 4-Br-BnIm

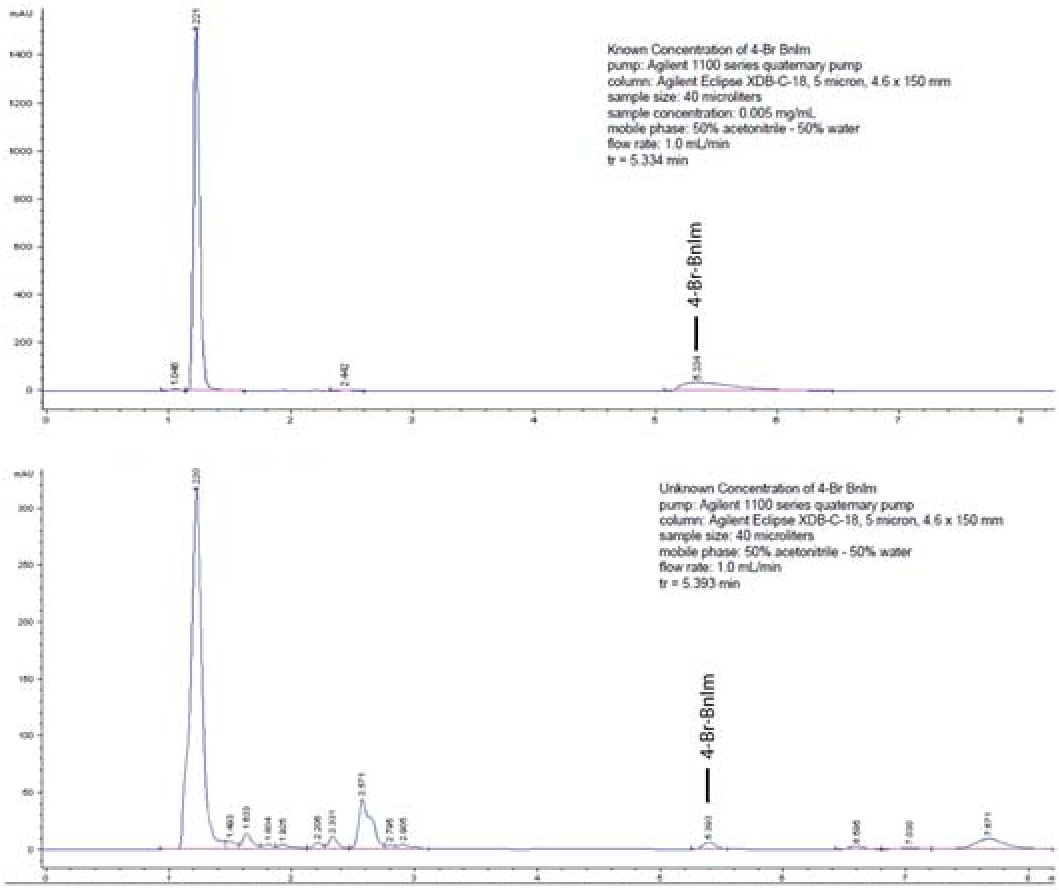

Upper trace represents one of the 4-Br-BnIm samples of known concentration that made up the standard curve. The lower trace represents an eye extract containing 4-Br-BnIm. The solvent front (MeOH) can be seen at 1.2 min and 4-Br-BnIm at ~5.3 minutes.

Extended Data Table 1: Crystallographic statistics

	**NΔ41 in complex with 4-Br-BnIm**
**Data collection**	
Space group	P 2_1 2_1 2_1
Cell dimensions	
a, *b*, *c* (Å)	65.237 84.42 95.473
a, *b*, *γ* (°)	90 90 90
Resolution (Å)	47.74 - 2.707[a] (2.803 - 2.707)
R_{merge}	0.121 (0.663)
$I/\sigma(I)$	22.11 (4.41)
$CC_{1/2}$	0.999 (0.954)
Completeness (%)	97.71 (83.38)
Redundancy	14.6 (14.9)
**Refinement**	
Resolution (Å)	2.7
No. reflections	217928 (21838)
R_{work} / R_{free}	0.1960 (0.2701)/ 0.2839 (0.3822)
No. atoms	
Protein	3307
Ligand/ion (specify/describe)	110
Water	38
B factors	
Protein	51.6
Ligand/ion	58.5
Water	37.3
R.m.s. deviations	
Bond lengths (Å)	0.009
Bond angles (°)	1.26

[a] Values in parentheses are for highest-resolution shell.
